# Stress-Induced Membraneless Organelles in Eukaryotes and Prokaryotes: Bird’s-Eye View

**DOI:** 10.3390/ijms23095010

**Published:** 2022-04-30

**Authors:** Anna S. Fefilova, Alexander V. Fonin, Innokentii E. Vishnyakov, Irina M. Kuznetsova, Konstantin K. Turoverov

**Affiliations:** 1 Laboratory of Structural Dynamics, Stability and Folding of Proteins, Institute of Cytology of RAS, 194064 St. Petersburg, Russia; a.fefilova@incras.ru (A.S.F.); alexfonin@incras.ru (A.V.F.); kkt@incras.ru (K.K.T.); 2 Group of Molecular Cytology of Prokaryotes and Bacterial Invasion, Laboratory of Cytology of Unicellular Organisms, Institute of Cytology of RAS, 194064 St. Petersburg, Russia; innvish@incras.ru

**Keywords:** stress, membraneless organelles, intrinsically disordered proteins, non-coding RNAs

## Abstract

Stress is an inevitable part of life. An organism is exposed to multiple stresses and overcomes their negative consequences throughout its entire existence. A correlation was established between life expectancy and resistance to stress, suggesting a relationship between aging and the ability to respond to external adverse effects as well as quickly restore the normal regulation of biological processes. To combat stress, cells developed multiple pro-survival mechanisms, one of them is the assembly of special stress-induced membraneless organelles (MLOs). MLOs are formations that do not possess a lipid membrane but rather form as a result of the “liquid–liquid” phase separation (LLPS) of biopolymers. Stress-responsive MLOs were found in eukaryotes and prokaryotes, they form as a reaction to the acute environmental conditions and are dismantled after its termination. These compartments function to prevent damage to the genetic and protein material of the cell during stress. In this review, we discuss the characteristics of stress-induced MLO-like structures in eukaryotic and prokaryotic cells.

## 1. Introduction

Cells of all organisms overcome the consequences of unfavorable external conditions throughout a lifetime. Stress has been suggested as one of the factors that determine the aging of the body [1]. At the cellular level, a link has been made between cellular stress resistance and delayed aging in several works on rodent-derived primary cell lines [2,3]. Moreover, it has been shown that stress is one of the leading factors driving cellular senescence, a phenomena which has been called stress-induced premature senescence (SIPS) [4]. For *Caenorhabditis elegans* it was demonstrated that mutations associated with prolonged longevity also promoted resistance to various forms of acute stresses, such as increased temperatures, heavy metals, UV radiation, and oxidizing agents [5]. A study performed on 58 healthy women found that both perceived and chronic stresses are associated with indicators of accelerated aging, such as oxidative stress, telomere length, and telomerase activity [6]. Thus, a body of collected evidence makes it possible to suggest that ability to overcome stressful conditions might be one of the key factors determining organismal longevity and that capacity to resist stress declines with age.

Cell fate during acute stress is determined by the balance between pro-survival and pro-apoptotic signaling [7]. The choice between the programmed cell death or survival depends on many factors defining the scale of the overall cell damage while the main mechanisms of stress-resistance include transcriptional and translational inhibition, selective initiation of transcription of heat shock proteins, etc. [8]. One of the mechanisms evolved by cells to combat stress are specific stress-induced membraneless organelles (MLOs).

MLOs are droplet-like structures that arise in the cell as a result of the liquid–liquid phase separation (LLPS) of biopolymers [9,10]. MLOs possess physicochemical properties characteristic of liquids and constantly exchange their contents with the intracellular environment [9,10]. These droplet-like condensates organize the intracellular space in both eukaryotes and prokaryotes, forming limited functional clusters of a unique composition. In addition to their structural role, MLOs are also involved in the regulation of signalling pathways, and failures in their function lead to the development of serious pathologies [10]. Many MLOs continuously found in cells under normal conditions, for example nucleoli, Cajal bodies, nuclear speckles, paraspeckles, histone locus bodies, P-bodies and others [11]. While others are transient and appear in response to stimuli, such as stress-induced MLOs.

The decisive role in the liquid–liquid phase separation leading to the formation of MLOs is provided by polymers lacking an ordered structure—intrinsically disordered proteins (IDPs) and RNA molecules.

IDPs and proteins that possess intrinsically disordered regions (IDPRs) make up an essential part of the proteome of both prokaryotes and eukaryotes. Thus, 25–30% of the proteins of the eukaryotic proteome and 15–20% of bacterial proteins are disordered, more than half of the eukaryotic proteins belong to IDPR, and among signal proteins, the share of IDPR reaches 70% [12]. One of the reasons of central roles of IDPs in MLOs formation is the conformational heterogeneity these proteins. An IDPs ensemble is a collection of protein molecules containing short, differently folded functional elements. This determines the multifunctionality of IDPs, the wide range of their partners, as well as their strong dependance on the external conditions. A slight impact can significantly change their properties and evolution over time. This allows us to consider IDPs/IDPRs and, consequently MLOs, as systems functioning “on the edge of chaos”—balancing between ordered and disordered states [13] The dysregulation of IDPs phase separation can induce the formation of highly toxic protein aggregates with an amyloid-like structure associated with the development of neurodegenerative diseases such as Alzheimer’s disease, amyotrophic lateral sclerosis, frontotemporal dementia, and other proteinopathies [14].

Long RNA molecules lacking a profound secondary structure also play an important role in the formation of MLOs. It has been unambiguously demonstrated that histone locus bodies, nuclear speckles, and nuclear stress bodies form around transcriptional centres of the corresponding RNA molecules. This suggests that RNA promote nucleation of at least of some MLOs, recruiting and accumulating resident proteins, increasing their local concentration, and promoting LLPS [15]. Interestingly, in the case of eukaryotic stress-induced MLOs discussed in this review, while core proteins of stress-induced MLOs are present in the cell permanently (including in the absence of stress), the MLO assembly starts with the emergence of free unstructured RNA. In case of cytoplasmic stress granules this process is driven by abundance of untranslated mRNA molecules released from polyribosomes due to inhibition of translation [16]. And in the case of A-bodies and nuclear stress bodies the MLO formation starts with stress-dependent activation of transcription of non-coding RNAs with subsequent MLOs assembling around RNA transcriptional loci [17,18].

As stated above, general properties of MLOs make it possible to consider these structures as open systems that represent a highly functional and dynamic soft matter. This property allows MLOs to quickly emerge/decompose in response to various intracellular signals and changing environmental conditions making them helpful stress-response regulators. Stress-inducible MLOs assemble both in the cytoplasm (stress granules) and in the nucleoplasm (A-bodies, nuclear stress bodies) of the eukaryotic cells with the onset of stress. Interestingly, biological condensates sensitive to stress with similar properties have been also described in bacteria (BR-bodies) [19], suggesting that this might be a universal survival mechanisms evolved earlier in the evolution. 

## 2. Stress-Induced Membraneless Organelles in Eukaryotes

Stressful conditions dramatically change cellular signaling pathways regulation. Inhibition of some and activation of other processes require simultaneous and interdependent coordination of thousands of new biochemical reactions. One of the main mechanisms of stress-response is the emergence of specific transient membraneless organelles as well as compositional changes in the previously formed ones. Here we consider membraneless organelles that appear in response to stress in the cytoplasm (stress granules) and nucleoplasm (A-bodies and nuclear stress bodies) of mammalian cells.

### 2.1. Stress Granules

The most studied MLOs formed in response to stress in eukaryotic cells are stress granules (SG). The formation of stress granules can be triggered by both exogenous and endogenous influences: temperature, oxidative and osmotic stress, UV radiation, impaired proteostasis, viral infection, and other causes [16,20]. The suggested function of SG is to promote cell viability under stressful conditions by preventing the formation of toxic aggregates of partially unfolded proteins and other biopolymers in the cytoplasm. This is achieved by isolating mRNA and various client proteins from the intracellular space into SGs [21]. This allows the cell to save resources necessary for the protein and mRNA degradation during stress exposure and synthesis of new molecules during stress recovery.

**Figure 1:**
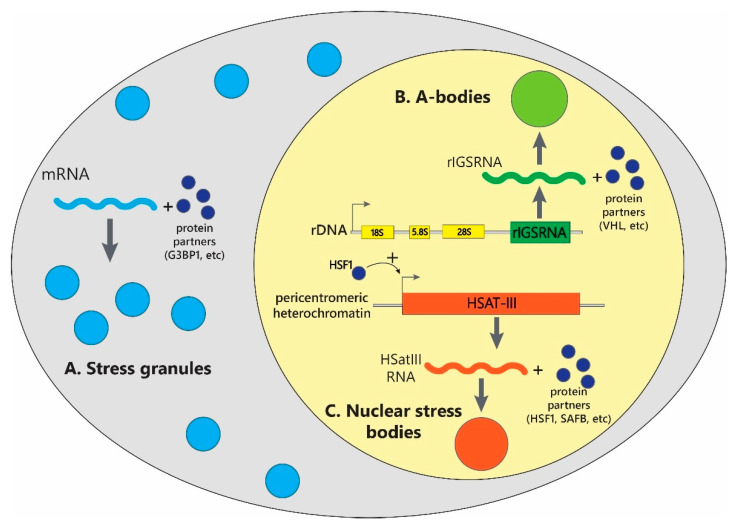
The schematic picture of formation of stress-induced MLOs in mammalian cells. The key players of stress-induced MLOs are shown. (**A**) Stress granules form in the cytoplasm as a result of stress-triggered release of untranslated mRNA from polyribosomes and its interaction with SGs core proteins, such as G3BP1 and others. (**B**) A-bodies form in the cell nucleus at the transcriptional loci of ribosomal intergenic spacer RNA (rIGSRNA), which transcription is activated upon stressful conditions from intergenic spacers of rDNA. Synthesized rIGSRNA interacts with VHL and other protein partners, leading to A-bodies formation. (**C**) Nuclear stress bodies assemble in the nucleus at the transcriptional sites of HSatIII RNA, located at the pericentromeric heterochromatin regions of chromosomes. HSatIII RNA transcription is promoted by HSF1 upon stress and via interaction with HSF1, SAFB and other protein partners drives condensation of nuclear stress bodies.

Cellular stress causes translation arrest typically via phosphorylation of translation initiation factors eIF2 and eIF4. The state-of-the-art research postulates that the formation of SGs is triggered by the dissociation of polyribosomes and mRNAs after abrupted translation, which is followed by the transition of SG scaffold proteins into the liquid droplet phase and recruitment of the free mRNA and other components into the condensates (Figure 1) [21,22,23,24]. The scaffold proteins of SG that undergo liquid–liquid phase transition in a presence of large amounts of free polyadenylated mRNA include partially intrinsically disordered G3BP1/2 (Ras GTPase-activating protein-binding protein 1/2) and TIA-1 (T-cell intracellular antigen-1) proteins containing RNA-binding RGG motifs (Figure 1). Other key components of SGs, such as transcription factors eI2F3, eIF4F, eIF4B, ribosomal 40S subunits, TIA-1-related (TIAR), polyadenylate-binding protein 1 (PABP1) and other proteins, are attracted to the resulting condensate due to a cascade of interactions.

The formation of SG takes place in several stages. Initially, a liquid-droplet condensate is formed, which, upon hardening, forms a low-dynamic central “core” of stress granules, around which client proteins form a more dynamic layer [25]. Thus, in mammalian cells, stress granules represent multiphase condensates consisting of a denser “core” and a less dense diluted phase [26]. Particularly important for the regulation of the functional activity of SGs is the composition of the dynamic layer. The constant exchange of the SGs content with the intracellular environment ensures timely regulation of the signaling pathways orchestrating the cell’s response to the onset and termination of stress. The dynamism of stress granules also allows these structures to be quickly dismantled after the stress ceases. Post-translational modifications play important role in regulation of SGs composition. The formation of SGs is stimulated by modifications that promote non-specific electrostatic interactions of modified proteins with negatively charged mRNA, such as o-acetylglucosamination, methylation, deacetylation, and dephosphorylation. On the contrary, phosphorylation of a number of SG scaffold proteins, in particular G3BP1, weakens the formation of stress granules [16].

Progression of several neurodegenerative diseases, including amyotrophic lateral sclerosis, Alzheimer’s disease, and frontotemporal dementia, is accompanied by dysregulation of the structure and properties of stress granules. Transformation of stress granules into toxic aggregates of amyloid fibrils is promoted by incorporation into them the disease-associated mutant forms of proteins, such as TIA-1, TIAR, FUS (RNA-binding protein fused in sarcoma), hnRNPA1 (heterogeneous nuclear ribonucleoprotein A1), TDP-43 (transactive response DNA binding protein 43 kDa), and PABP1 (polyadenylate-binding protein 1) [27,28,29,30,31,32,33].

Violation of the SGs degradation can also lead to the formation of ordered amyloid fibrils [34,35,36,37]. SGs breakdown can occur by an autophagosomal mechanism, even though SGs contain almost no ubiquitinated proteins [36]. The interaction between the autophagy regulator p62 and the expression product of the *C9orf72* gene attracts formed complexes to methylated arginine residues of SG IPDs and the subsequent binding of the stress granules to the LC3-II protein of the autophagosome membrane [24,26]. Mutations in *C9orf72*, in particular inclusion of hexanucleotide repeats, inhibit this process and promote the transformation of SGs into amyloid-like fibrils and development of neurodegenerative diseases [38]. The autophagosomal-independent mechanism of SGs post-stress decay is facilitated by chaperones and other proteins impairing the interaction between mRNA and SG scaffold proteins [39,40]. Importantly, that heat shock protein mRNA avoids inclusion into stress granules under any circumstances, probably due to extended 5’-UTR [41].

Stress granules also play a significant role in carcinogenesis [16]. The tumor microenvironment can be characterized by a high level of hypoxia, increased concentration of reactive oxygen species, and a low level of nutrients. These conditions promote the assembly of SGs and the activation of the cellular response, which contributes to the survival of the tumor cell. Etoposide and several other polychemotherapy drugs stimulate the formation of SGs, which may reduce the effectiveness of treatment. Moreover, the formation of SGs may promote the formation of metastases [16].

Currently, the reasons for the cytotoxicity of SG amyloid fibrils remain elusive. Possibly, the hardening of these organelles causes disturbances in the molecular exchange between SGs and the cytoplasm turning them into a physical trap for proteins regulating stress response and proteostasis whereby inhibiting their function [42]. As a result, levels of misfolded proteins elevate in the cell, to the point where protein quality system fails to effectively process them. This inevitably leads to the pathological degeneration of misfolded protein into cytotoxic oligomers and aggregates. Such model of ‘an indirect‘ stress granule cytotoxicity is largely consistent with the modern view on the reasons for the widespread involvement of IDPs into development of various proteinopathies [43]. Previously, it was believed toxicity of protein aggregation is attributed to the formation of mature amyloid fibrils. However, recent studies demonstrated that the precursors of amyloid fibrils, small oligomers, possess a significantly higher cytotoxicity [44]. Particularly, compared to mature amyloid fibrils, oligomers lacking an ordered tertiary structure have a significantly higher affinity for glutamatergic receptors, voltage-gated calcium channels and GM1-rich domains, and other membrane proteins [45,46,47]. Degradation of various cell membranes, primarily mitochondrial, by misfolded oligomeric aggregates causes a pronounced cytotoxic effect due to changes in membrane permeability, suppression of electron transport, and stimulation of the formation of high concentrations of reactive oxygen species [48,49,50].

### 2.2. A-Bodies

A-bodies are membraneless organelles that appear in the nucleolus in response to external stress and contain hundreds of proteins in the amyloid state (Figure 1) [17,51,52]. According to the modern concepts, A-bodies are formed in two stages. The stress effect on the cell induces the synthesis of long noncoding RNA, consisting of numerous dinucleotide repeats, from the so-called intergenic spacers of DNA encoding ribosomal RNA [51]. In mammals, ribosomal RNA is encoded by multiple tandem repeats, each of which is about 43 thousand bp long. Such repeats consist of genes encoding ribosomal RNA and a ribosomal intergene spacer (rIGS). For a long time, it was believed that rIGS are non-functional and represent fragments of non-transcribed (‘junk’) DNA [17]. However, recent studies have shown that several noncoding RNAs are transcribed from these DNA regions and are involved in the regulation of the level of rRNA expression, as well as in the formation of A-bodies. Such RNAs are called rIGSRNA (ribosomal intergenic spacer RNA). At the same time, the stress of different physical nature induces transcription of different regions of rIGS [53]. Thus, the stress effect on the cell induces the appearance in the cell nucleolus of extended negatively charged transcripts containing numerous dinucleotide repeats (CT)n/(AG)n [17]. An increase in the concentration of this type of rIGSRNA molecules, represented by sequences with a low degree of complexity causes the formation of biomolecular condensates, to which amyloidogenic proteins containing disordered positively charged regions rich in arginine and histidine residues are recruited due to electrostatic interactions. The presence of hydrophobic ACM (amyloid-converting motif) motifs in these proteins, provided that they are locally high in such organelles, creates conditions for the transformation of biomolecular condensates into a gel-like state and then into aggregates of amyloid fibrils [53]. This stage completes the maturation of A-bodies. It should be noted that stress of different physical nature causes not only the synthesis of various rIGSRNAs but also causes different protein compositions of A-bodies, which coincides by no more than 20% for bodies of different genesis [54]. The breakdown of A-bodies is initiated by the termination of stress and is carried out in an Hsp70/90-dependent manner [51]. At the same time, the proteins that make up these nuclear stress granules do not undergo degradation but change the topology of the polypeptide chain folding to the native conformation.

In fact, the reversible immobilization of hundreds of proteins under stress conditions during the formation of A-bodies causes a reorganization of the multiphase structure of the nucleolus, which, as is known, normally consists of a granular component, a fibrillar center, and a dense fibrillar center—condensates of various compositions with different surface tension [53]. It should be noted that, unlike A-bodies, aggregates of amyloid fibrils formed by mutant stress granule proteins are toxic to cells, however, the mechanism of such action of these structures is unknown. Perhaps in limiting the ability of A-bodies to contact with biomolecules from other phases of the nucleolus that the absence of toxicity of A-bodies is concluded.

### 2.3. Nuclear Stress-Bodies

Nuclear stress bodies (nSBs) are membraneless organelles specific for human and primate cells that are formed in the cell nucleus in response to thermal, chemical, proteotoxic, and some other types of stress (Figure 1) [18,55,56].

The size and number of nSBs depend on the type and duration of stress, but on average, several nSBs are formed in one cell, 1–2 μm in size [57]. nSB assembly occurs at transcription sites on the satellite DNA 3, which consist of tandemly organized repeats of nucleotide sequences located on several human chromosomes, but nSBs are mainly associated with chromosome 9 (locus 9q12). The main proteins that make up nSBs are the heat shock transcription factors HSF1 and HSF2; hnRNP proteins SAFB and hnRNPM; various mRNA splicing factors. The expression of satellite DNA 3 is triggered by the HSF1 transcription factor in response to stress and leads to a high local concentration of HSatIII (highly repetitive satellite III) noncoding RNA transcripts, which, together with HSF1, are the centers of nSBs nucleation (Figure 1).

In 2020, a study was published proving that nSBs are dynamic molecular condensates that form through liquid–liquid phase separation [56]. Moreover, it was shown that with an increase in the duration of stress exposure, the dynamics of HSF1 exchange with the nucleoplasm sharply decrease, which indicates the gradual hardening of HSF1-nSBs. Thus, after 16 h of incubation with proteasome inhibitor MG132, HSF1 mobility decreased by 40%, which was also confirmed by the addition of an inhibitor of liquid droplet condensates 1,6 hexanediol, which showed a decrease in the mobile fraction by 54% after 8 h of proteotoxic exposure [56]. The appearance of such insoluble HSF1-nSBs leads to an increase in cell sensitivity to apoptosis [56].

While HSF1 is required for the formation of nSBs, many other proteins are involved in their post-stress evolution. For example, various mRNA processing factors, such as splicing factors: SF2/ASF, SRp30, 9G8, were found in nSBs [18]. The SAFB protein is an hnRNP protein that binds to already formed stress granules via an RNA-binding domain [58] and becomes part of nSBs only in the presence of active HSatIII transcription. The predominance of HSF1 or SAFB proteins in nSB characterizes two functional stages of nSB formation that overlap in time: the HSF1 stage marks the onset of the stress response, and the amount of HSF1 rapidly decreases during the recovery period after the cessation of the stimulus. At the same time, the binding of SAFB to nSB begins an hour after exposure to mild thermal stress, reaching a maximum concentration 3 h after the end of the stress [58]. In a 2019 article, T. Hirose and others suggested that in addition to the canonical HSF1/SAFB nSBs, additional stress nuclear bodies are also formed in the cell as a result of the interaction of the hnRNPM protein and HSatIII [59]. However, it is possible that hnRNPM binding is not an additional type of nSBs, but the next functional step of nSBs.

At present, the biological function of nSBs is not fully understood. Initially, it was suggested that nSBs are transcription centers for HSF1 target genes, but this assumption was refuted when it was unambiguously shown that the localization of nSBs does not coincide with the centers of expression of heat shock proteins [60]. In addition, nSBs do not contain poly(A)-containing RNAs [58]. The positive role of nSBs in resolving post-stress deformations in the cell and enhancing the resistance of cells to temperature effects is obvious. For example, inhibition of HSF1 and SAFB expression by RNA interference has been shown to increase the sensitivity of HeLa cells to apoptosis [61]. In addition, it is known that SR proteins are translocated into nSBs as a result of stress, which suggests the role of nSBs in the post-stress regulation of mRNA splicing [62].

Studies show that the structural components of stress nuclear bodies, the HSF1 protein and the non-coding HSatIII RNA are involved in cell aging processes. Impairment of cellular proteostasis caused by the accumulation of molecular defects, such as structural damage of the proteins and the accumulation of misfolded peptides, is considered one of the main causes of the gradual aging of the body [63]. HSF1 is a transcription factor that regulates the functioning of chaperones, which normalize the tertiary and quaternary structure of misfolded proteins. An increase in the level of HSF1 expression in C. elegans tissues made it possible to significantly reduce the number of misfolded and prone to aggregation proteins, which extended the life of the animals [63]. At the same time, inhibition of HSF1 using the RNA interference reduced the lifespan of C. elegans [64,65] by 30–40%, and an elevation of HSF1 levels increased the lifespan by 22% in case of exogenous expression from the construct and by 40% in case of adding additional copies of the HSF1 gene to the nematode genome [65]. In addition, HSF1 is involved in mechanisms that control aging processes, such as insulin-regulated signaling (ILS) or signaling cascades triggered by dietary restriction (DR) [66]. Thus, there is a lot of experimental data that allows us to consider HSF1 as one of the key factors of life expectancy.

High levels of HSatIII expression, which is normally expressed only after stress exposure, were found in unstressed senescent human embryonic lung cells MRC5 at late passages [67]. A similar picture was observed in the primary cell culture obtained from patients diagnosed with the Hutchinson–Gilford syndrome, a genetic disease with clinical features of premature aging [68]. The Hutchinson—Gilford syndrome or progeria is characterized by a mutation in the gene encoding nuclear lamin A, which leads to deformation of the cell nucleus, as well as loss of heterochromatin. The study of epigenetic modifications in primary cells obtained from patients with progeria showed a loss of methylation of the pericentric regions of chromosomes. Interestingly, subsequent analysis of the expression of the main satellite DNAs (HSatIII and alpha satellite) by RT-qPCR and FISH showed that there were no changes in the expression levels of alpha satellite transcripts even after long-term cultivation. However, a significant increase in HSatIII levels expressed from chromosome 9 was found at medium and late passages in the unstressed cells [68].

In addition to senescent and progeria patient cells, stress-independent expression of HSatIII is also observed in cancer cells, suggesting that HSatIII transcripts play a role in the massive epigenetic genome rearrangements that occur in malignant cells [69].

Interestingly, HSF1 is not the only stress-sensitive transcription factor that activates HSatIII. It has been shown that, during hyperosmotic stress, HSatIII transcription depends on the osmosensitive transcription factor TonEBP/NFAT5 and does not depend on HSF1 [70]. TonEBP/NFAT5 regulates gene expression in response to osmotic stress and is vital for kidney function and protection from elevated salt and urea levels in the renal medulla [70]. At the same time, TonEBP/NFAT5 is also localized to nSBs, and knockdown of TonEBP/NFAT5 by siRNA prevents formation of nSBs in response to stress. The HSatIII sequence contains the putative TonEBP/NFAT5 binding site. These data suggest that nuclear stress bodies are part of the general cellular response to stress, responding to different stress-sensitive pathways depending on the type of exposure.

In addition to the stress response, HSatIII may also be involved in other processes. For example, exogenous expression in HeLa cells of another protein from the heat shock factor family, HSF2, whose increased expression is associated with the programs of embryonic development of the organism, activated HSatIII transcription in unstressed cells. This may mean that HSatIII is involved in developmental regulation [71]. This suggestion is supported by the fact that satellite DNA 3 is specifically expressed in the testes, which may indicate its role in the differentiation of primary germ cells [68,72].

Satellite DNA 3 sequences appeared relatively late in the evolutionary development and are not found in mammals other than primates [73]. Comparison of genetic material obtained from 25 primate species with 4 human cell lines showed that HSatIII arose suddenly about 16–23 million years ago [73]. In rodent cells, in response to stress, the formation of nuclear stress bodies or structures similar to them is not observed. However, flies have similar functional formations —omega speckles [74,75]. Omega speckles are nuclear micro formations that are present in large numbers in the cells of Drosophila fruit flies and contain many RNA-binding proteins. Their assembly depends on the long noncoding RNA Hsrω (heat shock RNA ω) transcribed from the 93D heat shock locus. When exposed to thermal stress, omega speckles coalesce at the site of Hsrω expression. It is assumed that they act as repositories for various factors involved in the mRNA processing and the RNA molecules themselves, contributing to a temporary post-stress inhibition of transcription [74]. Thus, omega speckles are potential functional analogs of nuclear stress bodies that emerged independently in the course of evolution [75].

## 3. Stress-Induced Membraneless Organelles in Prokaryotes

**Figure 2:**
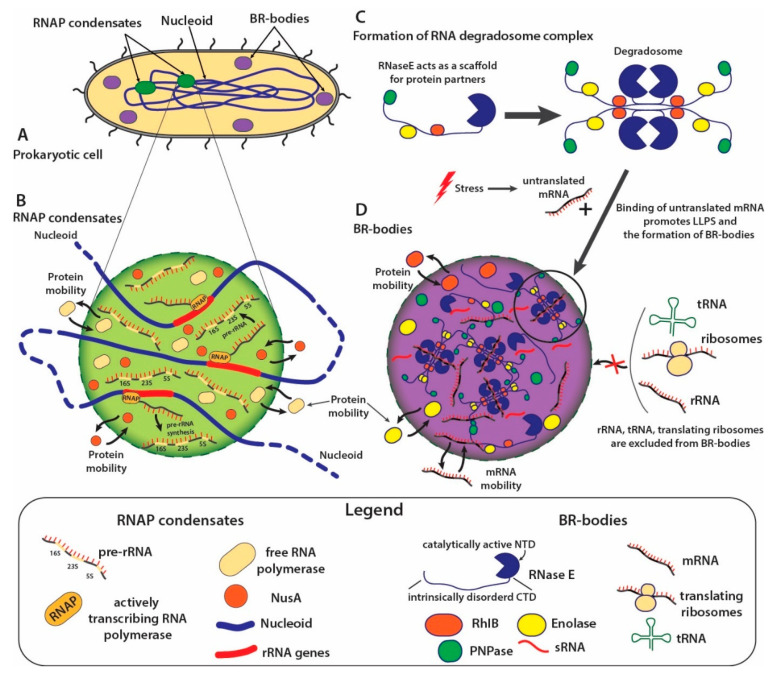
Schematic representation of RNAP condensates and BR-bodies formation in prokaryotic cells. (**A**) Prokaryotic cell with depicted relative localizations of nucleoid, RNAP condensates (green) and BR-bodies (violet). (**B**) RNAP condensates form via LLPS at the rRNA operons of nucleoid. They are associated with active rRNA transcription and contain active RNAP molecules, free RNAP molecules, synthesized pre-rRNA, as well as transcription anti-termination factor NusA. RNAP condensates demonstrate mobility of their content and other indicatives of liquid droplets. (**C**) RNase E contains intrinsically disordered C-terminal domain (CTD) containing multiple RNA-binding sites as well as interaction motifs for protein partners (RhIB, enolace, PNPase in *E. coli*). RNase E tetramers scaffold the RNA degradosome protein complex, which is a key component of BR-bodies. (**D**) BR-bodies formation in the bacterial cytoplasm is enhanced during stress by release of untranslated mRNA from the ribosomes. Upon interaction with untranslated mRNA RNase E tetramers undergo LLPS and form BR-bodies. BR-bodies demonstrate mobility of their content. LLPS nature of BR-bodies allows elimination of unnecessary translation-related molecules such as tRNAs, ribosomes, rRNA from BR-bodies.

Prokaryotic cells lack formalized cell nucleus and membrane organelles. At the same time, spatiotemporal coordination of hundreds and thousands of proteins in the cytoplasm of a prokaryotic cell requires highly complexed signaling mechanisms. It has been suggested that biomolecular condensates play a decisive role in the organization of the bacterial cytoplasm [19,76]. Thus, the absence of membrane organelles may be compensated by the existence of both large dynamic molecular machines such as the divisome [77], responsible for cell division, and membraneless organelles formed as a result of phase separation [19]. Recently, works started to appear that shed the light on the existing and potential LLPS-driven membraneless organelles present in prokaryotic cells (Table 1). Examples of such structures include RNA polymerase clusters (Figure 2A) [19], BR-bodies (Figure 2B) [78,79], PopZ microdomains and SpmX condensates in *Caulobacter crescentus* [80,81], ParABS protein system in *Corynebacterium glutamicum*, IbpA granular bodies in *Acholeplasma laidlawii,* single-stranded (ss)DNA-binding proteins (SSB) condensates [82], and many others (Table 1).

Large number of already published evidence supports hypothesis of general prevalence of LLPS formed condensates in signaling regulation of prokaryotes and a lot more supporting data may be expected in future. It has been shown that RNA polymerase clusters (RNAP) in *E. coli* forms clusters possessing properties typical for LLPS based structures, such as dynamic movement of their components [86] (Figure 2A). At the same time, RNAP foci are sensitive to hexanediol, which dissolves liquid-like compartments in eukaryotic cells. It has been demonstrated that the transcription anti-termination factor NusA, which undergoes LLPS in vitro and in vivo, is involved in the formation of the core of RNAP clusters [86] (Figure 2A). Importantly, RNAP clusters colocalize with 6 out of 7 the ribosomal RNA operons (rrn) in *E. coli* and are associated with active rRNA transcription. It indicates the presence of active RNAP molecules in these clusters [87], allowing to draw a line between prokaryotic RNAP condensates and nucleoli in eukaryotes.

Interestingly, RNAP compartmentalization was observed in *Bacillus subtilis* long before the emerging of the interest towards biomolecular condensates formed due to phase separation [88]. The exact mechanisms governing the formation of RNAP condensates in a bacterial cell have not yet been established. In particular, it is not known whether processing factors and modifying enzymes co-localize with bacterial RNAP condensates to promote co-transcriptional rRNA processing and ribosome assembly [19]. 

Prokaryotic cells are able to successfully withstand all kinds of stresses, from temperature [89] to oxidative stress [90], from drying [91] to high salt concentrations [61] etc. There is a possibility, that some prokaryotic analogs of eukaryotic stress granules could also be involved in stress response preventing the formation of insoluble protein aggregates subjected to partial or complete denaturation. One of the first responses to cellular stress is inhibition of translation, which leads to an accumulation of untranslated mRNA in the bacterial cytoplasm [78]. RNA degradosomes bind to the untranslated mRNA, that, triggers elevation of RNaseE local concentration, cross-interaction of the multiple multivalent domains and following BR-bodies formation via LLPS [78,79].

The so-called bacterial RNP bodies (BR bodies) (Figure 2B) are the closest functional analogue of eukaryotic stress granules described to date and also the most studied prokaryotic condensates. BR bodies are formed by RNase E endonuclease, which is essential for many bacteria species. RNase E possesses intrinsically disordered C-terminal domain (CTD) containing multiple RNA-binding sites as well as interaction motifs for protein partners. This allows RNase E CTD to act as a major scaffolding protein for RNA degradosomes, the active multi-protein complexes facilitating mRNA decay in bacteria. It was demonstrated that RNase E CTD undergoes phase separation in vitro and forms coalescing droplets in vivo [79]. Degradosomes condense into BR-bodies at the next level of compartmentation. Degradosome is formed by 4 RNase E proteins, recruiting other enzymes, which may vary depending on a particular organism. For the model gram-negative bacterium *Escherichia coli* the major canonical RNase E partners are DEAD-box helicase RhlB, the phosphorylytic exoribonuclease polynucleotide phosphorylase (PNPase) and the glycolytic enzyme enolase [92] (Figure 2B). Additionally, RNA chaperones and additional RNases are also common components of BR bodies [79]. However, the complete protein composition of BR bodies is still unknown.

BR bodies facilitate mRNA decay and utilization of mRNA decay fragments. Deletion of the intrinsically disordered C-terminal region of RNase E in *E. coli* or *C. crescentus* slows down the rate of mRNA degradation [93,94]. At the same time, BR bodies spacially separate mRNA decay processes from translation via physical elimination (due to selective permeabilization of the condensate) of RNAs with profound secondary structure, such as rRNA and tRNA, ribosomes, while engulfing long unstructured RNAs, such as untranslated mRNAs (Figure 2B) [93]. BR bodies have been found in many organisms containing RNase E dependent degradosomes: *Caulobacter crescentus*, *Sinorhizobium meliloti*, *Agrobacterium tumefacienes*, *E. coli*, and *Cyanobacteria* [19]. Also, similar formations were described for *Bacillus subtilis* [95] and *Helicobacter pylori* [96] in which degradosomes are scaffolded by RNase Y and RNase J respectively. Thus, the mechanisms of RNA transcription and degradation seem to be related to the formation of biomolecular condensates both in eukaryotic and bacterial cells.

Bacterial DEAD box ATPases (DDXs) involved in RNA metabolism have also been shown to form biomolecular condensates due to the presence of IDRs in their structure. Such ATPases, among other things, are involved in ribosome biogenesis, RNA turnover, and translation initiation. *E. coli* RhlB ATPase is able to interact with RNase E and is recruited into BR bodies [79]. The activity of this DEAD Box ATPase prevents BR bodies from hardening. Both eukaryotic and bacterial DDXs have been shown to promote phase separation in their ATP-bound form, while ATP hydrolysis induces compartment turnover and RNA release [85]. At the same time, DDXs control the flow of RNA in and out of phase-separated organelles, and thus may be able to create biochemical reaction centers that provide spatial and temporal control of various stages of RNA processing. 

Another example of stress-induced prokaryotic membraneless organelles may be the so-called granular bodies in mycoplasma *Acholeplasma laidlawii*, which numbers increase during heat shock [83,97,98] and unpublished data. These bodies include the small heat shock protein IbpA [83], which is capable of forming both large globular-type oligomers and fibrils [99]. IbpA interacts with many target proteins in the cell, which vary depending on the nature of stress [100]. It is possible that these granular bodies are equivalent of stress granules that allowing condensation of many cell proteins during adverse impact of external or internal factors. These proteins can be successfully refolded or utilized by intracellular proteases after stress is ended. As a positive argument for this view, association of heat shock proteins with stress-granules in the cytoplasm of eukaryotic cells can be considered [41]. In prokaryotic cells, the situation may be the same. However, discussions about the LLPS nature of IbpA granules are still speculative and require convincing experimental evidence.

A. *laidlawii* is one of the smallest currently known microorganisms [101] with highly reduced metabolic pathways. That would be reasonable to assume, that in the course of evolution it optimized cellular processes in order to most economically manage the available resources avoiding their scattering during unfavorable environmental conditions. The presence of stress-induced granular bodies resembling biomolecular condensates in such microorganism suggests the universal biological significance of membraneless organelles for cellular function.

In addition to the above mentioned membraneless formations, the so-called separation complex of the ParABS protein system responsible for the segregation of bacterial plasmids and chromosomes in bacteria is also formed by phase separation according to the liquid–liquid principle [84]. Liquid protein condensates are formed inside *E. coli* cells and during heterologous overexpression of intrinsically disordered eukaryotic proteins [102].

It was shown in [81] that phase separation in bacterial biomolecular condensates consisting of intrinsically disordered proteins can be facilitated by ATP depletion. The authors suggested that a diverse repertoire of such structures can play a significant role in the regulation of the activity of various enzymes in response to the metabolic state of bacterial cells. In addition, there is an opinion [103] that the formation and concentration of condensates can determine the total charge of globular proteins. In this study performed on supercharged *E. coli* proteins and nucleic acids, it was demonstrated that condensates are formed through electrostatic interactions between engineered proteins and RNA, and such condensates are dynamic and enrich only certain nucleic acids and proteins [103].

At same time, the phenomenon of phase separation can play an important role for some bacteria at the supracellular level. For example, the resistance of *Pseudomonas aeruginosa* cells to antibiotics increases when they are infected with the Pf4 prophage [104]. Liquid-crystal phage droplets form phase-separated occlusive compartments around rod-shaped bacteria, which leads to an increase in the survival of bacteria in biofilms [105]. Biophysical occlusion mediated by secreted filament molecules such as Pf4 may be a common survival strategy for bacteria in harsh environments.

## 4. Conclusions

Apparently, the transition of hundreds of proteins under stress conditions to the liquid-droplet phase with possible subsequent amyloidization is a universal mechanism for the preservation of a large number of proteins and RNA during the stress period. Such functional structures have been found in the cells of bacteria, fungi, plants, and mammals. As a rule, the proteins included in these structures contain domains of a low degree of complexity, i.e., are potentially predisposed to liquid–liquid phase transition and form droplets. Under certain conditions, these drops are transformed into functional fibrils. One of the main factors initiating the transition of proteins involved in the formation of stress-inducible membraneless organelles into the liquid droplet phase is high concentrations of RNA molecules, which not only act as a crowding agent that reduces the critical concentration of intrinsically disordered proteins necessary for their phase separation, but also determine the composition and properties of such membraneless compartments.

## Figures and Tables

**Table 1:** Examples of compartments formed or potentially formed by LLPS in prokaryotic cells.

Organelle	Key Players	Organism	Function
BR-body	mRNA, RNase E, Enolace, RhIB ATPase, PNPase, sRNA	*Escherichia coli* [78] *Caulobacter crescentus* [79] *Sinorhizobium meliloti, Agrobacterium tumefacienes, Cyanobacteria* [19]	mRNA decay. Formation is stimulated by cellular stress and following inhibition of translation
RNAP cluster	RNAP, NusA, rRNA operons	*Escherichia coli* [19]	rRNA transcription
IbpA granular bodies	IbpA	*Acholeplasma laidlawii* [83]	Stress-induced bodies involved in heat-shock response
ParABS protein system	ParA, ParB, ParS	*Corynebacterium glutamicum* [84]	plasmids and chromosome segregation
Bacterial DEAD box ATPases	Bacterial DEAD box ATPases, RhlB	*Escherichia coli* [85]	DEAD box ATPases have been shown to promote phase separation in their ATP-bound form. RhIB is a component of BR-bodies and involved in mRNA decay processes
SSB condensates	SSB	*Escherichia coli* [82]	Protection of single-stranded DNA during replication, recombination and repair
PopZ microdomains	PopZ	*Caulobacter crescentus* [80,81]	Polar organization during asymmetrical cell division
SpmX condensates	SpmX	*Caulobacter crescentus* [81]	Polar organization during asymmetrical cell division
